# Intra-tumor microbiome-based tumor survival indices predict immune interaction and drug sensitivity on pan-cancer scale

**DOI:** 10.1128/msystems.00312-25

**Published:** 2025-06-25

**Authors:** Yan Gao, Haohong Zhang, Dongliang Chu, Kang Ning

**Affiliations:** 1 Key Laboratory of Molecular Biophysics of the Ministry of Education, Hubei Key Laboratory of Bioinformatics and Molecular-imaging, Center of AI Biology, Department of Bioinformatics and Systems Biology, College of Life Science and Technology, Huazhong University of Science and Technology 12443, Wuhan, Hubei, China; 2 Geneis Beijing Co. Ltd., Beijing, China; The University of Hong Kong, Hong Kong, Hong Kong

**Keywords:** tumor microbiome, survival analysis, machine learning

## Abstract

Growing research evidence indicates a substantial influence of the intra-tumor microbiome on tumor outcome. However, there is currently no consistent criterion for identifying the association of microbes with tumor progression and response to treatment across various types of cancer. In this study, we concentrate on the intra-tumor microbiome and develop the Tumor Microbiome Survival Index (TMSI), a measure indicative of cancer patient survival risk. Our indices revealed notable distinctions between two stratified risk groups for each of the 10 cancer types and could precisely predict patients’ overall survival. For each type of cancer, our findings unveiled two distinct gene expression profiles and shed light on the varying patterns of immune and stromal cell enrichment between the two risk groups. Additionally, we noted that the high-TMSI group exhibited substantially elevated IC50 values for a number of drugs, indicating that individuals in the low-TMSI group might experience superior therapeutic effects from chemotherapy. These findings illuminate the complex dynamics between the tumor microbiome, the patient’s immune reaction, and medical outcomes, thus shedding light on microbiome-based personalized therapeutic interventions.

**IMPORTANCE:**

This work presents the Tumor Microbiome Survival Index (TMSI), a crucial innovation. It stratifies cancer patients into risk groups across 10 cancer types, accurately predicting survival. By uncovering distinct gene expression and immune/stromal cell patterns, it deepens understanding of tumor complexity. The finding of altered drug sensitivity in different TMSI groups offers insights for personalized chemotherapy. Overall, it paves the way for microbiome-targeted cancer therapies and enhanced patient prognostication.

## INTRODUCTION

Cancer is the second-leading cause of death worldwide, accounting for approximately 2.6 million deaths (1, 2). Traditionally, cancer has been regarded as a disease originating from alterations in the genetic makeup of human beings (3, 4). Consequently, there has long been a recognized connection between tumor gene expression and cancer outcomes (5–8). However, while genetic markers have been instrumental in advancing our understanding of cancer progression, their clinical applicability is limited. Relying solely on genetic markers provides an incomplete comprehension of cancer biology (9).

A tumor is not merely a cluster of cancer cells; it is a heterogeneous assembly of infiltrating and resident host cells, secreted factors, extracellular matrix, and possibly microbes, all of which influence prognosis (10). This complexity is further compounded by the genetic heterogeneity that tumors often exhibit, even within the same cancer type. Such heterogeneity arises from factors including mutations, chromosomal rearrangements, and epigenetic modifications (9). This heterogeneity can lead to variations in gene expression patterns among different tumors and even within different regions of the same tumor. Therefore, integrating multi-omics data is essential for prognostic analysis (11, 12).

The tumor microbiome, a complex and diverse community of microbes that inhabit human tumors and adjacent tissue, has gained increasing attention for its potential role in cancer biology (13, 14). Although there is still debate regarding the extent of the relationship between microbes and cancer, a growing body of evidence supports the involvement of the tumor microbiome in various types of cancer. Poore et al. (15) recently developed a computational workflow that utilizes two orthogonal microbial detection pipelines to obtain high-quality microbial abundances from high-throughput sequencing data of human tumors. The tumor microbiome has been shown to play certain functions in the development (16), progression (17), and response to treatment (18) for various types of cancer. The relationship between the tumor microbiome and patient survival is the subject of ongoing research, as some studies (19, 20) have suggested that the microbiome of a tumor may impact patient survival. For example, *Malassezia globosa* has been linked to a higher risk of mortality in breast cancer patients, underscoring the potential impact of specific microbes on patient outcomes (20). Therefore, the intra-tumor microbial signal could serve as a potential candidate for accurately prognostic prediction. Nevertheless, a consistent criterion for capturing these survival-related microbes in different tumors is lacking, and the mechanisms through which microbiota contribute to shaping the molecular properties of tumors and influencing clinical outcomes remain poorly understood (21–23).

Given the growing recognition of the tumor microbiome’s role in cancer prognosis, we introduce the Tumor Microbiome Survival Index (TMSI), a survival risk score based solely on the tumor microbiome. Applied to a cohort of cancer microbiome data set from Voest et al. (24), our index demonstrated significant differentiation of two risk groups in 10 cancer types and accurately predicted the 1-year, 2-year, and 4-year overall survival (OS) rates. The TMSI has also been validated on an external pancreatic cancer data set (22). We then analyzed the molecular-level differences between the two risk groups using paired gene expression and DNA methylation data, focusing on potential crosstalk between the host immune system and the tumor microbiota. Our findings revealed distinct patterns of immune cell and stromal cell enrichment between the two risk groups, with certain microbiota potentially regulating immune gene expression by influencing DNA methylation levels. Furthermore, we observed significantly higher IC50 values for several drugs in the high-risk group, suggesting that patients in the low-risk group may derive greater benefit from these medications. These results shed light on the intricate interplay between the tumor microbiome, host immune response, and clinical outcomes, thereby offering new avenues for personalized therapeutic interventions.

## MATERIALS AND METHODS

### Construction of the TMSI model

The TMSI model was constructed using microbiome data from tumor tissues of 3,052 samples across 10 types of cancer. To identify prognostic-related bacteria, we first performed univariate analysis using the proportional hazards model implemented in the “survival” R package (version 3.6-4). Bacteria with *P*-values less than 0.05 were selected as candidates for further evaluation.

To refine this selection, we applied the random survival forest (RSF) model from the “randomForestSRC” R package (version 3.3.0). Specifically, microbial features that passed the univariate Cox regression filter (*P* < 0.05) were ranked based on their importance in the RSF model. The top 50 most important microbes were retained for the next stage, except in cases where fewer than 50 microbes met the univariate significance threshold, in which case all were carried forward.

Next, the LASSO method was employed to further refine the selection, retaining only microbes with non-zero coefficients. These microbes were then incorporated into a final Cox regression analysis, where their corresponding coefficients were used to construct the TMSI score.

The parameters for the random survival forest analysis are specified as follows: the number of trees, ntree, is set to 2,000; the minimum node size, nodesize, is 20; the data set used is rf_data; the number of splits, nsplit, is 20. The method for handling missing data is set to na.impute, and tree-level error estimation is enabled by tree.err. The importance of variables will be assessed as importance is set to TRUE. The random seed for reproducibility is set to 999.

The algorithm ranked each bacterium according to importance, and we selected the 50 most important bacteria for subsequent analysis. Through the “glmnet” R package (version 4.1-8), these 50 bacteria were used as inputs for the least absolute shrinkage and selection operator (LASSO) Cox regression model, ultimately screening out the significant bacteria. Based on the abundance of the corresponding bacteria and the Cox coefficients of the patients, the Tumor Microenvironment-based Immune Score Indices (TMSIs) for each patient can be calculated using the algorithm of the inner product of matrices. The calculation method has been publicly announced as follows:


$$TMSI=\;\sum_{i=1}^{n}\left(coefficient\;i\right)*\left(abundance\;i\right)$$

In this formula, *n* represents the total number of independent prognostic tumor microbiome biomarkers selected by univariate and multivariate Cox regression for predicting patient survival. The “coefficient *i*” refers to the regression coefficient of the *i*th tumor microbiome biomarker in the multivariate Cox regression, and “abundance *i*” refers to the microbial abundance of the *i*th tumor microbiome biomarker.

As is standard in Cox-based prognostic modeling (25, 26), the TMSI score is computed by multiplying the abundance of each selected microbe by its corresponding coefficient *i*, obtained from LASSO-Cox regression. The coefficient of each microbe is determined by LASSO-COX regression, which first uses Lasso regression for variable selection and then constructs a Cox regression model to analyze the prognostic impact. Therefore, the final coefficient *β* obtained is directly related to the hazard ratio (HR), where $HR=e^{\beta }$. If the coefficient *β* obtained from LASSO-COX regression is positive, it indicates that the feature (tumor microbiota abundance) is associated with a higher risk; if the coefficient is negative, it indicates a lower risk.

We found that tumor microbiome prognostic markers independently affect patients’ OS in all cancers. Utilizing the optimal cutoff value determined by the “surv_cutpoint” function, patients within these 10 cancer types were stratified into TMSI-high and TMSI-low groups. To evaluate the predictive performance of TMSI in estimating patients’ OS, Kaplan-Meier curves and ROC curves were employed.

### Evaluation and validation of the TMSI model

In order to improve the forecasting accuracy of 1-year, 2-year, and 4-year survival rates across various cancers, we amalgamated clinical characteristics (including age, subtype, gender, and stage) with TMSI to develop a prognostic nomogram model. This was accomplished utilizing the “rms” (v6.8.0) package. The performance of the model was assessed using time-dependent Receiver Operating Characteristic (ROC) curves and the concordance index (C-index).

In our study of the cohort of 10 types of cancer, we specifically validated pancreatic adenocarcinoma (PAAD) using the PRJNA542615 data set. This data set includes 16S rRNA sequencing data from 43 tumor tissue samples with known survival status. Microbial community composition for these data sets was generated using Qiime2 (27). In the discovery cohort, we identified 10 genera of bacteria to construct the TMSI model, but four of them were not detected in the validation cohort. To address this issue, we employed an interpolation method, using the average abundance of these bacteria from the discovery cohort as the estimated abundance for the corresponding bacteria in each sample of the validation cohort. Subsequently, we used the bacterial coefficients determined from the discovery cohort to calculate the TMSI for each sample in the validation cohort.

### Integrated analysis of the tumor microenvironment and the tumor microbiome

To describe the tumor microenvironment landscape of patients in TMSI-high and TMSI-low groups, matching tumor RNA-seq data of these two subtypes across cancers was introduced. The differentially expressed genes (DEGs) between these two groups were calculated by the DESeq2 (v 1.44.0) package using the raw count of RNA-seq with the thresholds (|log2FoldChange|  <  1 and adjust *P* < 0.05). Gene lists for Kyoto Encyclopedia of Genes and Genomes (KEGG) and Gene Ontology (GO) were sourced from the MSigDB database (https://www.gsea-msigdb.org/gsea/msigdb/). Subsequently, Gene Set Enrichment Analysis (GSEA) was conducted utilizing the “clusterProfiler” (v4.10.1) package. Immune-related DEGs were annotated and mapped to GO terms and KEGG pathways using the ImmPort database (https://www.immport.org/home). The prognostic significance of these immune-related DEGs was evaluated through univariate Cox regression analysis and Kaplan-Meier survival curves. Furthermore, we explored the underlying correlation between immune-related DEGs and circulating microbial prognostic markers by Spearman correlation. Additionally, immune cell infiltration in tumors was assessed using the “xCell” (v1.1.0) package. Finally, expression levels of immune checkpoint molecules such as PD-1/PD-L1 and CTLA4 were compared between the two groups.

### Mediation analysis

We employed the mediation (v4.5.0) package to perform mediation analysis, with the alteration in microbial abundance as the independent variable (*X*), the change in gene expression as the dependent variable (*Y*), and the change in gene methylation as the potential mediator variable (*M*). The beta value indicates the strength of the influence of the independent variable (*X*) on the mediator variable (*M*) and the mediator variable (*M*) on the dependent variable (*Y*). Specifically, the beta value is a regression coefficient that reflects the linear relationship and its direction between the independent and mediator variables, as well as between the mediator and dependent variables. In standardized regression, the beta value represents the correlation between the independent and dependent variables. Standardized regression coefficients facilitate comparisons across different units or variables by eliminating the impact of different variable scales. Moreover, when the beta value is statistically significant (*P* < 0.05), it signifies that the independent variable can effectively predict variations in the dependent variable, implying a significant impact of the independent variable on the dependent variable.

### Drug sensitivity

We set out to evaluate the predictive significance of interactions between the tumor microenvironment and the immune system in forecasting the success of immunotherapy and chemotherapy treatments. The Tumor Immune Dysfunction and Exclusion (TIDE) score, accessible at http://tide.dfci.harvard.edu/, served as the tool for predicting immunotherapy response across various cancer types. Furthermore, to ascertain the sensitivity of patients to chemotherapy drugs, we leveraged the “oncoPredict” package (v1.2) in R. This tool facilitated the computation of the half-maximal inhibitory concentration (IC50) for each patient, thereby enabling us to predict their likely response to chemotherapy agents.

### Statistical analysis

All statistical analyses and graphical representations were conducted using R software (R version 4.3.1). The Wilcoxon rank-sum test served to compare continuous variables between the TMSI-high and TMSI-low groups. A significance threshold of *P* < 0.05 was adopted to ascertain statistical significance.

## RESULTS

### Cohorts and workflow

**Fig 1:**
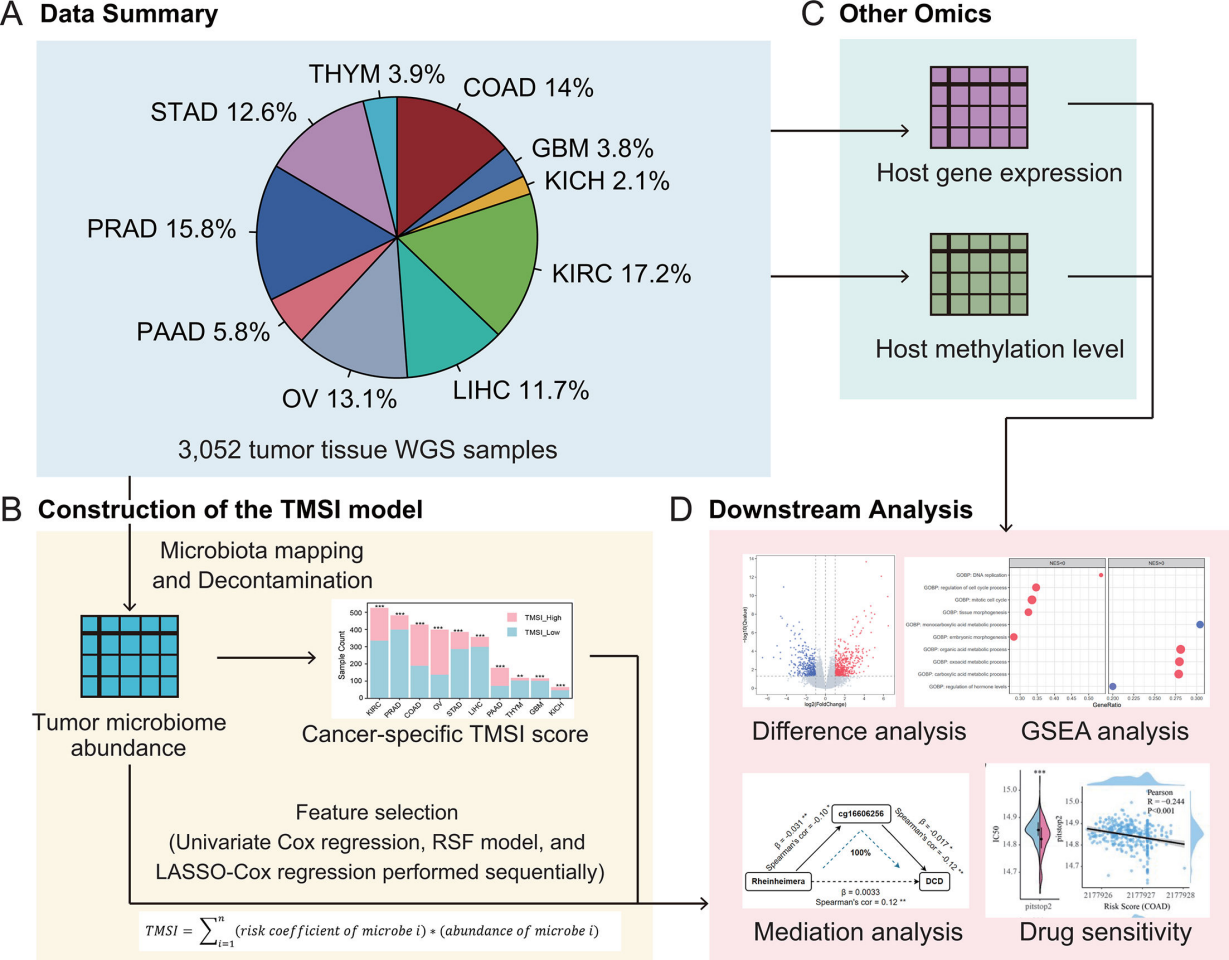
Workflow for a comprehensive analysis of the tumor microbiome across various cancer types. (**A**) Data summary. Our discovery cohort includes 3,052 tumor tissue samples across 10 cancer types. (**B**) Development of the TMSI model based on tumor microbiome profile data. (**C**) Integration of hosts’ RNA-seq data and methylation array data. (**D**) Subsequent downstream analysis.

Our discovery cohort encompasses 3,052 tumor tissue samples across 10 cancer types, with paired omics data comprising microbiome profile, RNA-seq, and methylation array data. Microbiome profile data were obtained from Poore et al. (28), subjected to Kraken2 and PathSeq microbial profiling pipeline. After rigorous decontamination to eliminate potential contaminants, the data set was refined to 1,041 microbial genera. RNA-seq and methylation array data were sourced from The Cancer Genome Atlas (TCGA) via the Genomic Data Commons (https://portal.gdc.cancer.gov/). The meta-information accompanying these data sets includes clinical parameters such as age, gender, and cancer stage, as well as survival data encompassing survival status and time (Fig. 1A). For validation, we used the PRJNA542615 PAAD data set as validation cohort, which includes 16S rRNA sequencing data from 43 tumor tissue samples with known survival status (22).

For the association analysis and comparison, we initially identified tumor microbes with independent prognostic significance using univariate cox regression, RSF model, and LASSO-Cox regression (Fig. 1B). The TMSI score for each patient was then calculated by combining the abundance of OS-related microbes and the coefficients from the LASSO-Cox regression. Based on their TMSI scores, patients were divided into TMSI-high and TMSI-low groups across each type of cancer. To explore the molecular underpinnings of TMSI score variation, we incorporated the gene expression data and methylation data from the host’s tumor tissue and examined the tumor gene expression profiles to investigate their relationships with TMSI scores (Fig. 1C). Ultimately, we evaluated the TMSI score’s capability to predict responses to cancer therapies, emphasizing its potential to forecast the success of chemotherapy (Fig. 1D).

### Construction of the TMSI model

**Fig 2:**
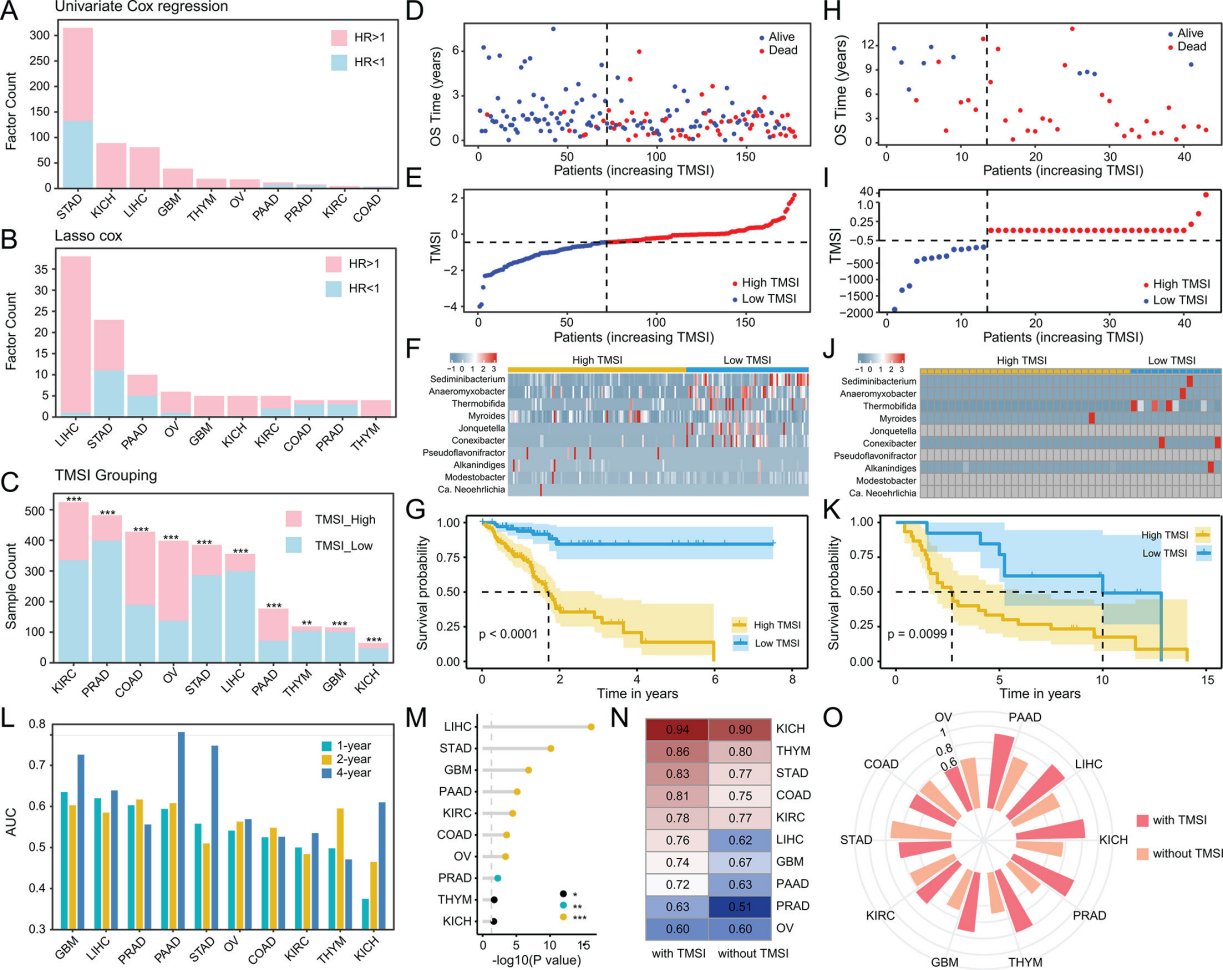
Construction, evaluation, and validation of the TMSI model to predict the prognosis of patients with 10 types of cancer. (**A**) Bar graph showing candidate microbial factors significantly associated with OS by univariate Cox regression analysis. (**B**) Bar graph of microbial factors independently impacting OS by Lasso-Cox regression analysis. (**C**) Significance markers above the bars display the *P*-values calculated by the Log-rank test between the TMSI-high and TMSI-low groups. (**D–G**) Performance of the TMSI model for PAAD in the discovery cohort. (**H–K**) Performance in the PAAD validation set. (**D, H**) Each point in the scatter plot represents the survival status and survival time of a patient. The horizontal coordinates are the patients ranked from the lowest to highest according to their TMSIs. (**E, I**) Based on the risk score of each point in the scatter plot representing one patient, they are divided into TMSI-high and TMSI-low groups. (**F, J**) The heatmap demonstrates the expression levels of the ten TMSI model bacteria in patients. (**G, K**) The Kaplan-Meier curves show the OS time of the two risk groups of patients. (**L**) The Area Under the Curve (AUC) of the Receiver Operating Characteristic (ROC) curve shows the performance of TMSI in predicting the 1-year, 2-year, and 4-year OS rates. (**M**) The lollipop chart illustrates whether TMSI has an independent impact on prognosis after adjusting for clinical characteristics (including age, subtype, gender, and stage). (**N**) The *C*-index value of the nomogram model based on clinical factors with or without the inclusion of TMSI. (**O**) The AUC values of the time-dependent ROC curves show the performance of the nomogram models with or without the inclusion of TMSI in predicting the 4-year OS probabilities of the patients.

First, we employed univariate Cox regression analysis to identify genera significantly associated with the OS of cancer patients across 33 cancer types. Following feature selection, only 10 cancer types were identified as having significant survival-related features (Fig. 2A; Table S1). This analysis revealed both risk factors, with HR > 1 (*P* < 0.05), and protective factors, with HR < 1 (*P* < 0.05). Subsequently, we utilized the random survival forest model to select the 100 most important bacteria and further performed LASSO-Cox regression analysis to delineate genera that independently impact OS (Fig. 2B). In all 10 types of cancer, we successfully identified genera that independently affect OS. These results formed the basis of the TMSI model, which calculates a patient’s mortality risk by multiplying the abundance of OS-related genera with the coefficients derived from the LASSO-Cox regression. The optimal cutoff value for the TMSI was determined using the “surv_cutpoint” function, thereby stratifying patients into high-TMSI and low-TMSI groups. The Kaplan-Meier curve and the Log-rank test revealed that patients in the high-TMSI group had shorter OS compared to those in the low-TMSI group (Fig. 2G and K; Fig. S1A through I), with the number of samples in each group and the *P*-value of the Log-rank test displayed in Fig. 2C. Notably, the microbes contributing to the TMSI varied significantly across cancer types, with only a few, such as *Candidatus Neoehrlichia*, *Collimonas*, *Lachnoanaerobaculum*, *Paraclostridium*, *Pyramidobacter*, and *Subdoligranulum*, appearing in two cancers (Table S2). This variability suggests that the prognostic relevance of specific genera may be context-specific, highlighting the need for further validation across a broader range of cancers.

The inverse relationship between TMSI and OS reflects the underlying principle that TMSI quantifies mortality risk based on the weighted abundance of OS-related genera. Positive coefficients correspond to risk-associated genera (HR > 1), while negative coefficients indicate protective genera (HR < 1). A lower TMSI suggests a microbiome composition enriched with protective genera and/or depleted of risk-associated genera, potentially fostering a more favorable tumor microenvironment and ultimately contributing to longer OS.

### Validation and evaluation of the TMSI model

To validate the TMSI model on an external cohort, we used the PRJNA542615 data set, which includes 16S rRNA sequencing data from 43 tumor tissue samples. In the discovery cohort, we identified 10 genera of bacteria to construct the TMSI model, but 4 of them were not detected in the validation cohort. To address this issue, we employed an interpolation method, using the average abundance of these bacteria from the discovery cohort as the estimated abundance for the corresponding bacteria in each sample of the validation cohort. Subsequently, we used the PAAD TMSI formula from the discovery cohort to calculate the TMSI for each sample in the PAAD validation set. Using the TMSI formula established from the PAAD samples in the discovery cohort, the TMSI was calculated for each sample in the PAAD discovery cohort (Fig. 2D) and for each sample in the PAAD validation set (Fig. 2H). Based on the optimal cutoff value determined from the PAAD data set in the discovery cohort, both the PAAD data set in the discovery cohort (Fig. 2E) and the PAAD validation set (Fig. 2I) were divided into low TMSI and high TMSI groups. Excluding the four bacteria not detected in the validation set, the other bacteria used to construct the PAAD TMSI had a similar abundance distribution in the discovery cohort (Fig. 2F) and the validation set (Fig. 2J). Additionally, Kaplan-Meier analysis showed that patients with a higher TMSI had worse OS in both the discovery cohort (Fig. 2F) and the validation set (Fig. 2J).

The TMSI exhibited robust performance in predicting 1-year, 2-year, and 4-year OS rates. Specifically, the AUC values for predicting the 1-year OS rates for GBM and LIHC were 0.635 and 0.620, respectively. Moreover, the AUC values for predicting the 4-year OS rates for PAAD, STAD, and GBM were 0.781, 0.748, and 0.726, respectively (Fig. 2L). To ascertain whether the TMSI could function as an independent prognostic indicator, we conducted a multivariate Cox regression analysis based on the TMSI and clinical characteristics, including age, subtype, gender, and stage. The analysis results demonstrated that the TMSI independently affected OS in 10 cancers (Fig. 2M).

Subsequently, we formulated nomogram survival models for these cancers integrating clinical factors, both with and without the TMSI, to predict the 1-year, 2-year, and 4-year OS probabilities for patients. The nomogram models incorporating the TMSI exhibited higher *C*-index values compared to those without the TMSI (Fig. 2N). The time-dependent ROC curves in predicting the 1-year (Fig. S1J), 2-year (Fig. S1K), and 4-year (Fig. 2O) OS probabilities of the patients suggest that the TMSI enhanced the accuracy of prognostic prediction.

In summary, we have identified microbial genera significantly associated with the prognosis of cancer patients and validated the effectiveness of the TMSI model constructed from these genera in predicting prognosis. The TMSI shows promise as a valuable tool for enhancing the accuracy of prognostic models.

### The stratification of TMSI score exhibits distinct patterns of gene expression

**Fig 3:**
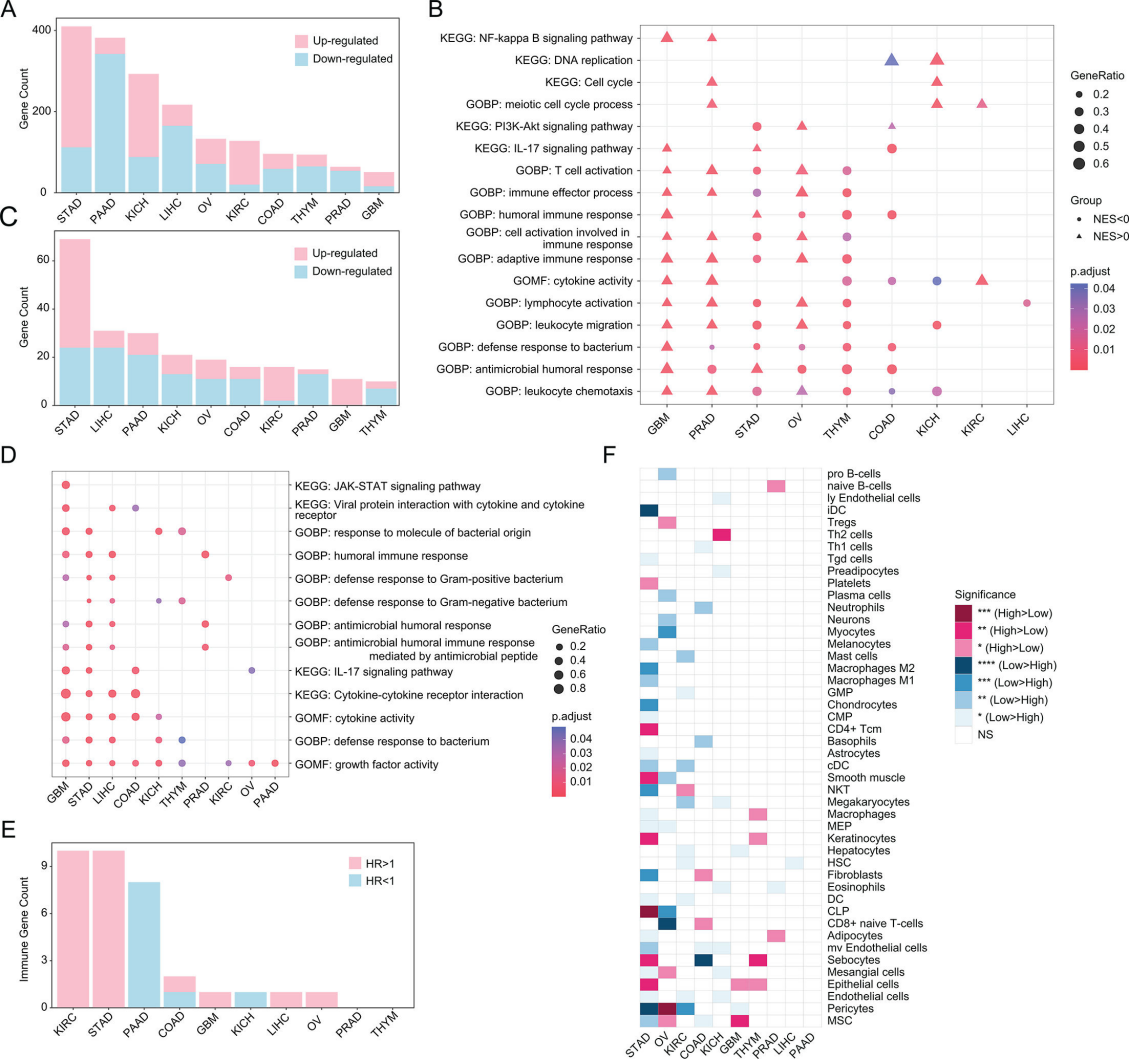
Elucidation of the tumor immune microenvironment through TMSI model. (**A**) Bar chart summarizes the differentially expressed genes (DEGs) (P.adj < 0.05 and |log2 FC| > 1) in tumor tissues between the TMSI-high group and the low-TMSI group as determined by DESeq2 analysis. (**B**) Bubble chart displays the frequently occurring KEGG pathways and GO terms identified by GSEA enrichment analysis. NES < 0 represents gene sets are up-regulated in low-TMSI. (**C**) Bar chart tallies the immune-related DEGs in tumors within the TMSI-high group compared to the TMSI-low group. (**D**) GO and KEGG enrichment analysis based on the immune-related DEGs within each cancer type. (**E**) The number of survival-related and immune-related DEGs identified through univariate cox regression and log-rank test across each type of cancer. (**F**) Differences in xCell scores of immune cells and stromal cells between TMSI-high group and TMSI-low group (F, ****, *P* < 0.0001; ***, *P* < 0.001; **, *P* < 0.01; *, *P* < 0.05).

Then we examined the tumor gene expression profiles and investigated their relationships with TMSI scores. We discerned differentially expressed genes (DEGs) (adjusted *P* < 0.05 and |log2 fold change| > 1) between TMSI-high and TMSI-low groups. Across 10 cancers, an asymmetrical distribution in the count of upregulated and downregulated DEGs was evident (Fig. 3A). Gene Set Enrichment Analysis (GSEA) outcomes consistently revealed the enrichment of canonical proliferative gene sets, such as “Cell Cycle,” “DNA Replication,” and “Mitotic Cell Cycle Process” in the TMSI-high group. However, we only observed immune-related gene sets such as “Defense Response to Bacterium” and “Antibacterial Humoral Response” exhibited predominant enrichment in the low-TSMI group in STAD, THYM, and COAD (Fig. 3B).

Interestingly, certain immune gene sets such as “Lymphocyte Activation” and “Cell Activation Involved in Immune Response,” alongside immune pathways like the “IL-17 Signaling Pathway,” demonstrated enrichment in the TMSI-high group in GBM, PRAD, and OV. This observation may stem from an immune-suppressive state within the corresponding tumor microenvironment, wherein tumor cells exert control over immune cells. Despite apparent gene expression activity, the resultant effect tends toward immune suppression rather than activation. These findings underscore the intricate interplay between tumor TMSIs and the tumor immune microenvironment. High TMSI scores may correlate with increased immune suppression and cell proliferation, while low TMSI scores may be associated with enhanced immune activation and engagement of metabolic pathways. Understanding these mechanisms may provide insights into immune regulation within tumors and support the development of novel therapeutic strategies.

To further explore immune-related DEGs, we used data from the ImmPort database to identify immune-associated Differentially Expressed Genes (DEGs) (P.adj < 0.05 and |log2 FC| > 1) within tumor tissues, distinguishing between the TMSI-high and TMSI-low cohorts. The distribution of upregulated and downregulated immune-related DEGs across various cancers paralleled that of the overall DEGs (depicted in Fig. 3A and C). Additionally, Gene Ontology (GO) and Kyoto Encyclopedia of Genes and Genomes (KEGG) enrichment analyses revealed the involvement of these immune-related DEGs in “Humoral Immune Response,” “Defense Response to Bacterium,” “Cytokine-Cytokine Receptor Interaction,” and signaling pathways intimately linked to immune system function (Fig. 3D). Further analysis involved identifying immune-related DEGs significantly correlated with overall cancer patient survival via univariate Cox regression and Kaplan-Meier survival analysis (Fig. 3E). In the histogram, pink bars represent immune-related DEGs deemed risk factors for survival (HR > 1; *P* < 0.05), while blue bars signify those considered favorable factors (HR < 1; *P* < 0.05). Moreover, tumor immune infiltration analysis unveiled varying degrees of immune cell infiltration between tumors with high and low TMSIs (Fig. 3F). Tumors with high TMSI scores exhibited increased immune surveillance, suggesting stronger immune responses. However, this may also imply potential resistance to immune attacks through mechanisms such as upregulation of immune checkpoint molecules, contributing to a higher risk of poor survival outcomes.

### TMSI predicts the chemotherapy efficacy

Microbes play a crucial role in modulating the responses of patients to cancer therapeutics, underscoring their significant influence on treatment efficacy. Numerous studies have affirmed that patients with elevated expression levels of PD-1 or CTLA4 tend to derive greater benefits from immunotherapy. In our study, we observed significant differences in the expression of the immune checkpoint molecule CTLA4 between TMSI-high and TMSI-low groups in COAD (Fig. S3A), and similarly, significant differences in the expression of the immune checkpoint molecule PD-1 between TMSI-high and TMSI-low groups in LIHC (Fig. S2B). In both cases, the expression was higher in the TMSI-low group compared to the TMSI-high group, suggesting that patients in the TMSI-high group may have a higher risk of survival because they are less likely to benefit from immunotherapy. Subsequently, we conducted TIDE (Tumor Immune Dysfunction and Exclusion) immunotherapy response assessments to explore the predictive value of TMSI for the outcomes of immunotherapy. In 10 types of cancer, no significant differences were found between the TMSI-high and TMSI-low groups, indicating that TMSI has limited capability in predicting the efficacy of immunotherapy (Fig. S3).

**Fig 4:**
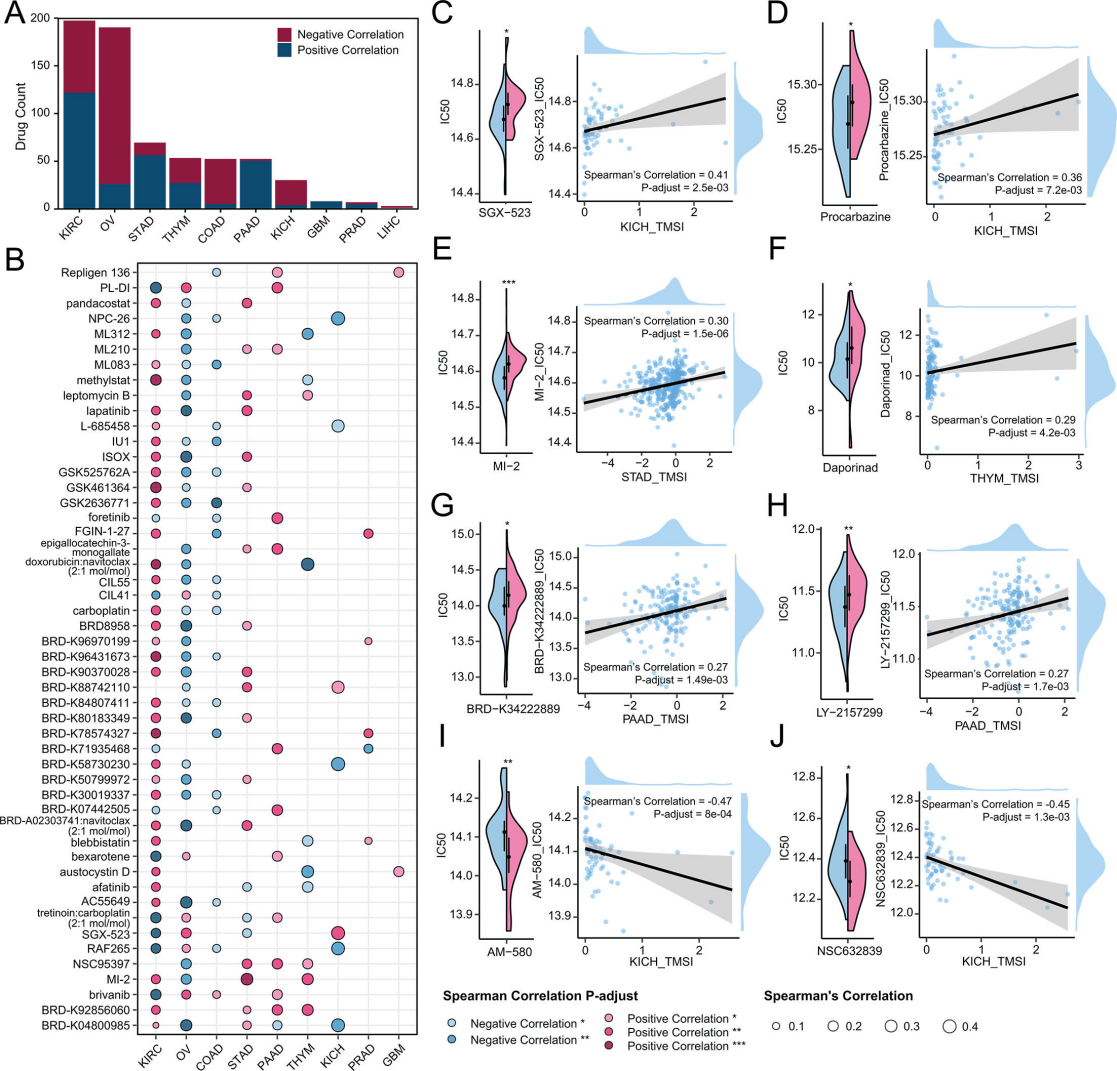
Evaluation of the predictive efficacy of TMSI models in chemotherapy. (**A**) Statistics of drugs significantly different between the TMSI-high group and the TMSI-low group as well as correlated with the TMSI across 10 cancers. (**B**) Presenting the top 50 high-frequency drugs significantly correlated with TMSIs across each cancer. (**C–J**) Violin plots and correlation plots of the comparison in IC50 values between the TMSI-high group and the TMSI-low group. The blue part represented the TMSI-low group, and the pink part represented the TMSI-high group in violin plots. **P < 0.01; *P < 0.05.

**Fig 5:**
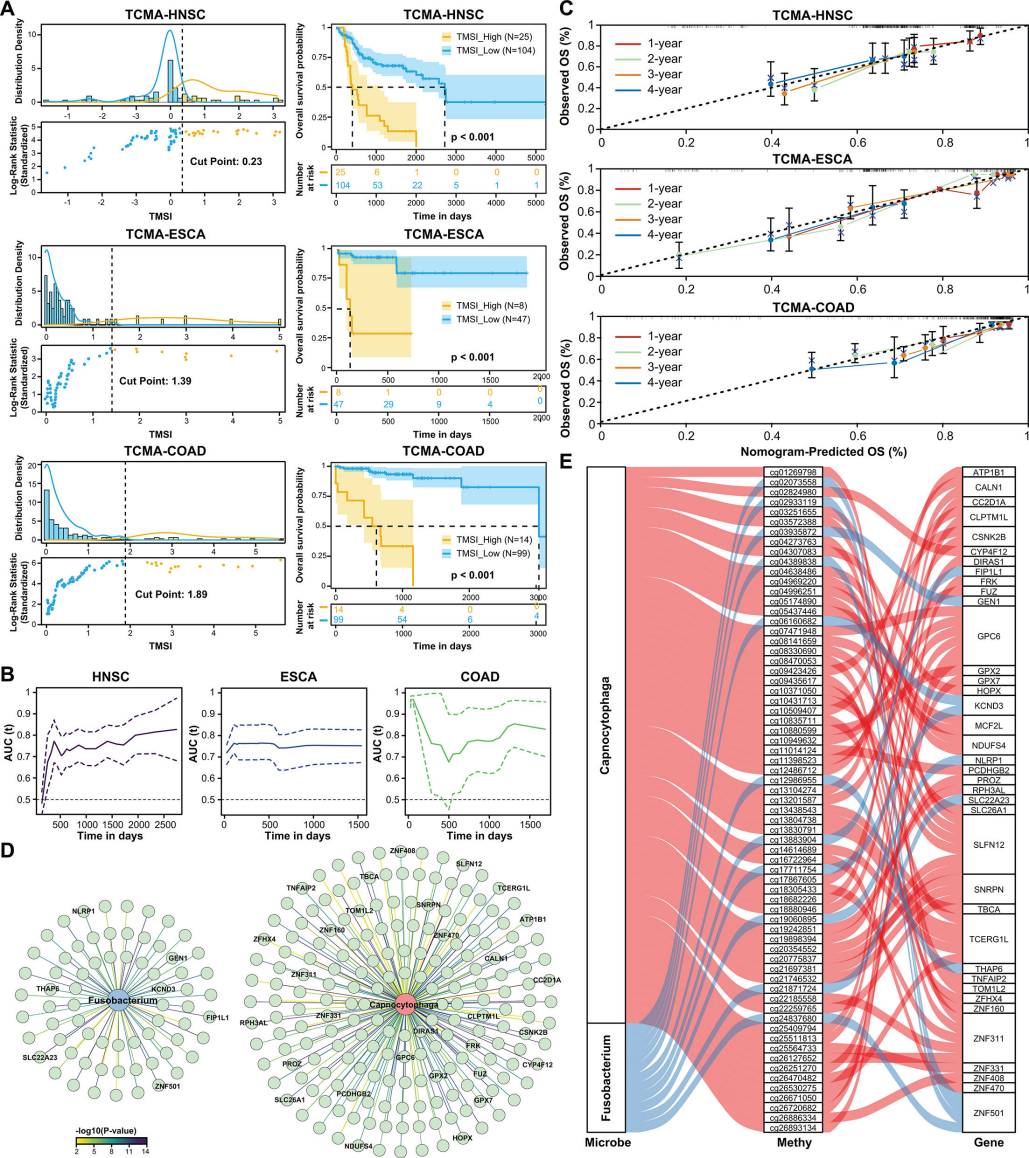
Validation of the TMSI model and mediation analysis on the TCMA data set. (**A**) Cut-point selection plots and Kaplan-Meier survival curves for three cancer types in the TCMA data set. (**B**) Time-dependent ROC curves evaluating the predictive performance of the TMSI score across three cancers. (**C**) Calibration curve assessing the agreement between predicted and observed outcomes for the nomogram integrating the TMSI score with clinical indices. (**D**) Correlation network illustrating the associations of *Fusobacterium* and *Capnocytophaga* with their correlated genes. (**E**) Sankey diagram depicting the mediation analysis pathway linking microbial presence, methylation probes, and host gene expression.

To further elucidate the relationship between TMSI and sensitivity to chemotherapy drugs, we employed the “oncoPredict” software package to forecast the IC50 values for each drug across different cancers. Substantial disparities in chemotherapy responses were evident between high and low TMSI groups across 10 cancers, with STAD displaying the most drugs with significant differences (Fig. 4A). Among the top 50 high-frequency drugs significantly correlated with TMSI in each cancer type, a greater number of drugs exhibited IC50 values significantly higher in the high TMSI group compared to the low TMSI group (Fig. 4B). Specifically, IC50 values of SGX−523 in KICH (Fig. 4C), Procarbazine in KICH (Fig. 4D), MI−2 in STAD (Fig. 4E), Daporinad in THYM (Fig. 4F), BRD−K34222889 in PAAD (Fig. 4G), and LY−2157299 in PAAD (Fig. 5H) were significantly higher in the high TMSI group compared to the low TMSI group, indicating higher resistance to these chemotherapy drugs among patients in the high TMSI group. Conversely, IC50 values of AM−580 in KICH (Fig. 4I) and NSC632839 in KICH (Fig. 4J) were significantly higher in the low TMSI group compared to the high TMSI group, suggesting potential benefits for patients in the high TMSI group from these drugs (Table S3).

### Validation of the TMSI model and microbiome-host interaction analysis

In this study, we obtained tumor microbiome data from a previous investigation of cancer microbiomes (28). However, it is important to note that the decontamination procedures applied to tumor microbiome data have been a subject of ongoing debate (29, 30). Concerns have been raised regarding the incomplete removal of host sequences, which could lead to the misannotation of these sequences as tumor-associated microorganisms. Several genera identified as survival-related microbes for TMSI model construction are typically associated with environmental contexts. While these issues do not diminish the overall utility of the model—since the identified microbial signals remain statistically significant with respect to survival outcomes—they may impact the interpretability of the model in terms of its biological significance.

To address these concerns, we validated the TMSI model using The Cancer Microbiome Atlas (TCMA), a curated collection of decontaminated microbial compositions from five cancer types (31). The TMSI model was successfully constructed for three cancer types (HNSC, ESCA, and COAD), and the TMSI score demonstrated significant differences in survival outcomes (log-rank test, *P* < 0.05) for each cancer type (Fig. 5A). The time-dependent ROC curve consistently exceeded 0.7 across all time points, further confirming the robustness of the TMSI score for survival prediction (Fig. 5B). Additionally, we integrated the TMSI score with other clinical indices to construct a nomogram, and the calibration curve of the nomogram showed high consistency, validating its clinical applicability (Fig. 5C).

We conducted an in-depth exploration of the dynamic interplay between the host’s immune system and microbial inhabitants on TCMA data set (31), scrutinizing the mechanisms by which the tumor microbiota might modulate gene expression through the alteration of gene methylation patterns. We observed that *Fusobacterium* and *Capnocytophaga* in ESCA exhibited significant with cancer-related genes like NLRP1 (32) and ZFHX4 (33) (Fig. 5D). Emerging evidence has suggested that microbes can influence host biology by modifying epigenetic states such as methylation (34, 35), prompting us to hypothesize that tumor microbiota regulate gene expression by modifying DNA methylation patterns. To test this, we employed mediation analysis to assess whether tumor microbes indirectly influence gene expression by altering methylation status. Notably, we noticed that *Capnocytophaga* regulated the expression of PCDHGB2 by enhancing methylation at the cg11014124 site (Fig. 5E; Table S4). Cg11014124, located on the shelf of CpG islands, is linked to PCDHGB2, a gene associated with gastrointestinal diseases. Our analysis suggests that *Capnocytophaga* enhances methylation at this site, potentially regulating PCDHGB2 expression. As an oral commensal with pathogenic potential, *Capnocytophaga* may contribute to tumor microenvironment remodeling through epigenetic modifications, linking microbial activity to host gene regulation in cancer.

We further applied the TMSI method to a re-annotated tumor microbiome data set across three cancer types: HNSC, BLCA, and BRCA. The TMSI scores effectively stratified patients into two groups with significantly different survival outcomes (Fig. S4A through C), and time-dependent AUC analyses demonstrated robust predictive performance, with values predominantly above 0.7 (Fig. S4D through F). Correlation analyses of survival-associated microbes and immune-related genes revealed a distinct pattern in HNSC, where *Micrococcus* and *Pseudoramibacter* abundances positively correlated with most immune genes, while *Klebsiella*, *Pyramidobacter*, and *Streptococcus* abundances showed negative correlations; no significant patterns were observed in BLCA or BRCA (Fig. S4G).

## DISCUSSION

In this study, we introduced the Tumor Microbiome Survival Index (TMSI), a model designed to evaluate the prognostic value of microbiome signatures for various cancer types. By analyzing microbiome profiles across 10 different cancers, we identified specific tumor-associated microbes that are significantly correlated with patient survival. Previous studies have shown increasing evidence that microbial signatures can be leveraged to predict cancer prognosis, offering insights into tumor heterogeneity and disease progression (21, 23).

Our approach extends beyond establishing correlations by delving into potential mechanistic insights. The TMSI model shows initial promise as a prognostic tool, suggesting that microbial signatures may influence survival outcomes through modulation of gene expression, possibly via alterations in the methylation landscape. Gene Set Enrichment Analysis further supports this by highlighting distinct functional pathways associated with TMSI groups. The enrichment of proliferative pathways in the high-TMSI group suggests that certain microbial profiles may contribute to tumor progression, while the presence of immune-related pathways in the low-TMSI group, observed in only a few cancers (e.g., STAD, THYM, COAD), points to a potential protective role of the microbiome in specific contexts. Such dual roles of the microbiome—in either promoting or inhibiting tumor progression—have been observed in studies examining microbial influence on tumor immunity and inflammation (36).

These findings underscore the complexity of microbiome-tumor interactions and suggest that the TMSI could illuminate not only survival differences but also variations in tumor biology and immune dynamics. Our analysis hints at interactions between TMSI microbiome signatures and the tumor immune microenvironment, particularly the humoral immune response, as well as potential implications for chemotherapy sensitivity (37). Emerging evidence supports the idea that microbiota can modulate chemotherapy efficacy by shaping the immune landscape or metabolizing drugs. However, these associations are predominantly observed in a limited subset of cancers, and broader applicability remains to be established. We propose that these findings reflect an early step toward understanding the complex interplay between microbial communities, immune responses, and treatment outcomes, rather than definitive conclusions applicable to all cancers. This limitation highlights the importance of expanding the scope of future studies to include additional cancer types and larger cohorts.

For patients with cancers such as KICH, PAAD, STAD, or THYM, the TMSI emerges as a candidate prognostic biomarker with potential to inform personalized therapeutic strategies, including chemotherapy sensitivity. Yet, these insights are derived from a limited subset of cancers, and their broader applicability remains uncertain without further evidence. The variability in microbial contributions across cancer types, as reflected in our functional analyses, reinforces the need for careful interpretation and additional investigation.

Although the TMSI model has demonstrated efficacy in predicting the prognosis of patients with various cancers, there are certain limitations that warrant further discussion. A major limitation is the lack of sufficient publicly available data sets to fully validate our findings; thus, we have only assessed the predictive capacity of TMSI for survival risk in patients with PAAD. Moreover, although our cross-validation results support the predictive utility of TMSI, we acknowledge that contamination remains a potential confounder. Thus, further validation across larger, well-controlled cohorts and experimental verification are essential to confirm these findings and disentangle true biological signals from potential artifacts. Additionally, while we explored the indirect influence of tumor microbes on gene expression through methylation, this represents a preliminary finding requiring deeper mechanistic investigation.

In summary, our research offers new perspectives on the development of cancer and the intricate interactions between the immune system and microbial communities. Our joint analysis revealed potential interactions between TMSI microbiome signatures and tumor immune microenvironment, especially the humoral immune response. Moreover, TMSI has guiding significance in the use of chemotherapy treatments, potentially leading to more personalized therapeutic strategies for cancer patients.

### Highlights

- The TMSI model generates a survival risk score based on the intra-tumor microbiome.
- The TMSI model predicts survival rates accurately across multiple cancer types.
- Molecular-level analysis highlights distinct immune interactions with tumor microbes.
- High-TMSI groups showed altered drug sensitivity, implying potential differences in treatment response.

## Data Availability

All code is available at https://doi.org/10.6084/m9.figshare.27094327.v1.

## SUPPLEMENTAL MATERIAL

Supplemental Figures msystems.00312-25-s0001.docx Figures S1 to S4.

Table S1 msystems.00312-25-s0002.xlsx Number of retained features at each selection step.

Table S2 msystems.00312-25-s0003.xlsx Survival-associated microbes used in the construction of the TMSI model for each cancer type.

Table S3 msystems.00312-25-s0004.xlsx Chemotherapy response prediction results for each cancer type.

Table S4 msystems.00312-25-s0005.xlsx Mediation analysis results on the TCMA data set.

ASM does not own the copyrights to Supplemental Material that may be linked to, or accessed through, an article. The authors have granted ASM a non-exclusive, world-wide license to publish the Supplemental Material files. Please contact the corresponding author directly for reuse.
