# Supplementary material for: Intra-tumor microbiome-based tumor survival indices predict immune interaction and drug sensitivity on pan-cancer scale
Source: mSystems. 2025 Jun 25;10(7):e00312-25. doi: 10.1128/msystems.00312-25 (PMC12282056; doi:10.1128/msystems.00312-25)
Supplement: Supplemental Figures — Figures S1 to S4. [file msystems.00312-25-s0001.docx]

**Supplementary Figures**

**
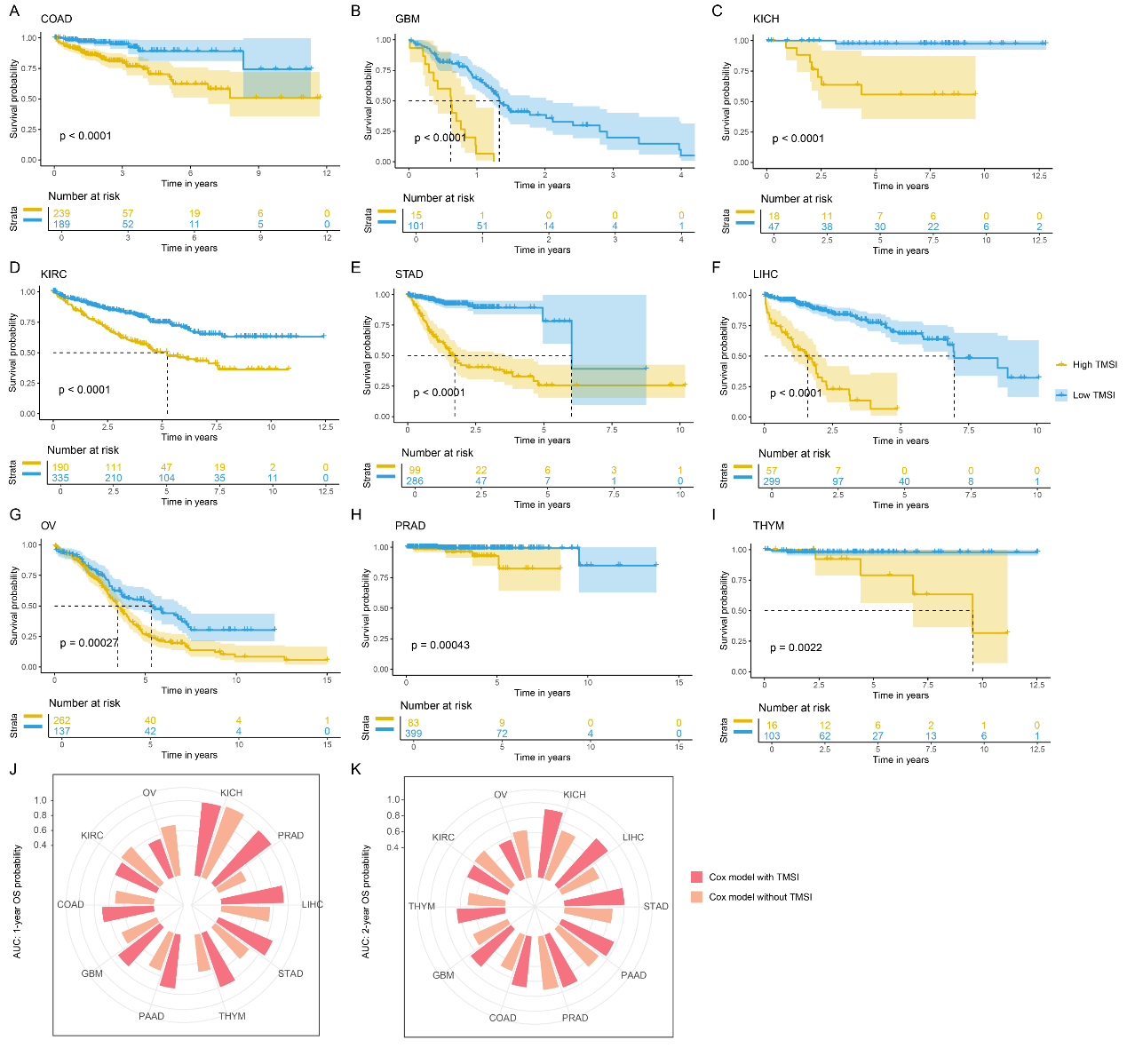
**

**Supplementary Figure 1. A-I** The Kaplan-Meier curve illustrates the overall survival associated with the TMSI in the other 9 cancers. The P-value was determined by the log-rank test between the TMSI-high group and the TMSI-low group. The horizontal axis represents the survival time (time in days) of cancer patients, and the vertical axis represents the survival probability of cancer patients. The orange curve represents patients with TMSI-high in various cancers, and the blue curve represents patients with TMSI-low in various cancers. The threshold for dividing the high and low TMSI groups in each type of cancer was calculated using the “surv_cutpoint” function from the “survminer” R package. **J-K** The area under the curve (AUC) values of the time-dependent ROC curves demonstrates the performance of the nomogram models with or without the inclusion of the TMSI for predicting the **J** 1-year and **K** 2-year overall survival (OS) probabilities of the patients.


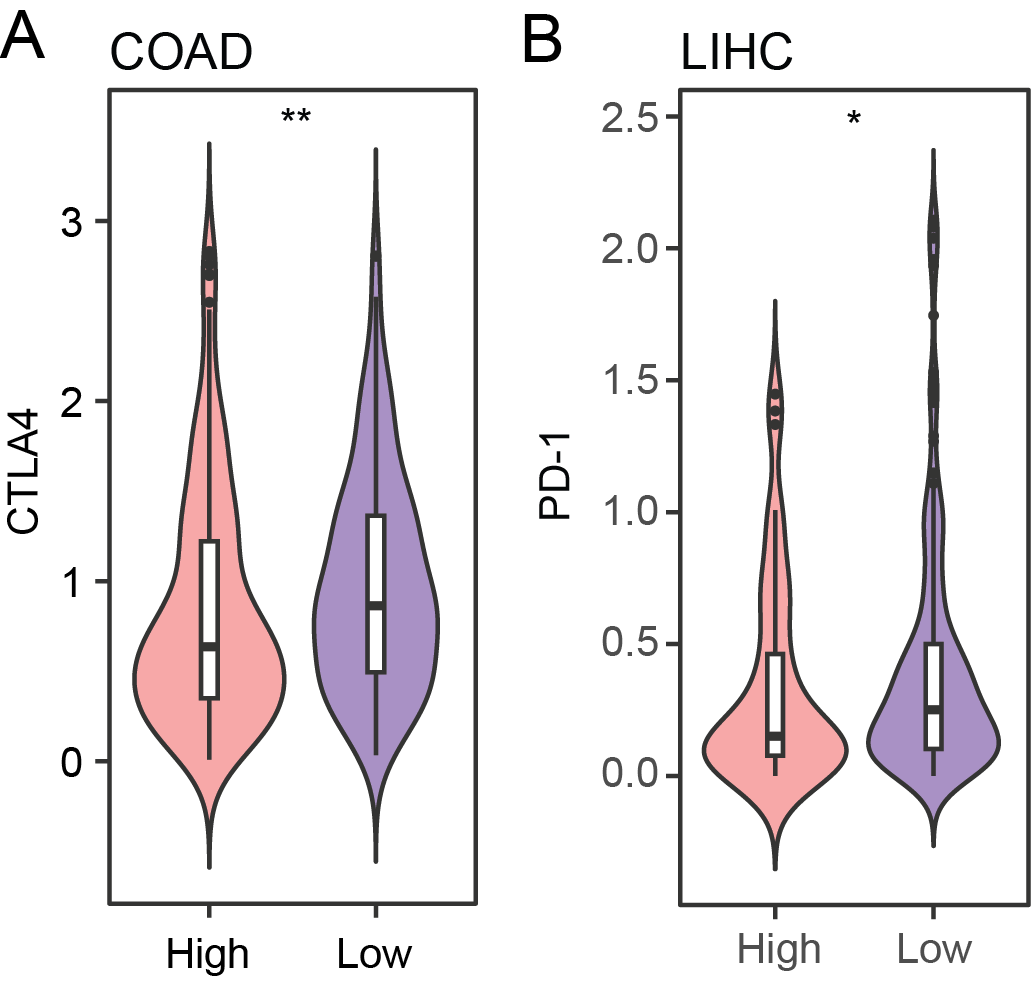


**Supplementary Figure 2. A.** CTLA4 expression levels in COAD between TMSI-high and TMSI-low groups (**, p<0.01; *, p<0.05). **B.** PD-1 expression levels in LIHC between TMSI-high and TMSI-low groups (**, p<0.01; *, p<0.05).


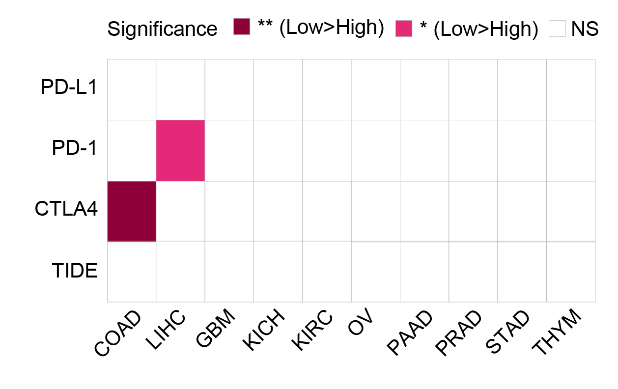


**Supplementary Figure 3.** Differences in PD-1/PD-L1 expression levels, CTLA4 expression levels and the TIDE scores between the TMSI-high group and the TMSI-low group.

**
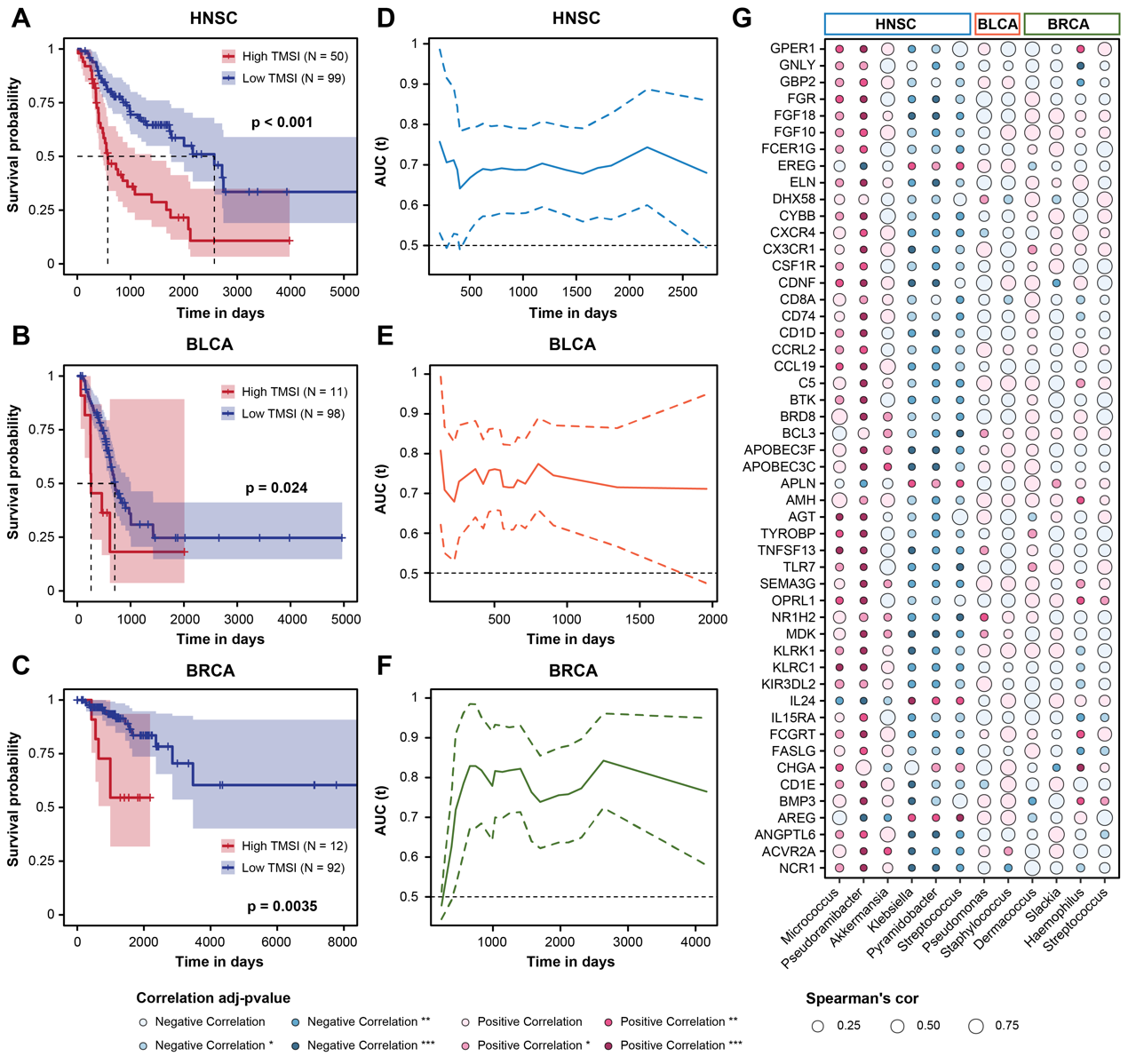
**

**Supplementary Figure 4. TMSI Analysis and Microbial-Immune Correlations Re-Annotated Dataset.** **A–C.** Kaplan-Meier survival curves for tumor patients stratified by TMSI scores in HNSC, BLCA, and BRCA, demonstrating significant survival differences between high- and low-TMSI groups. **D–F.** Time-dependent AUC plots for TMSI in HNSC, BLCA, and BRCA, with AUC values predominantly exceeding 0.7. **G.** Correlation heatmap illustrating relationships between survival-associated microbiota and immune gene expression in HNSC, with *Micrococcus* and *Pseudoramibacter* showing positive correlations, and *Klebsiella*, *Pyramidobacter*, and *Streptococcus* displaying negative correlations with most immune genes.
